# Effects of cranioplasty with customized 3D titanium mesh plates on postoperative complications and neurological outcomes following traumatic brain injury or stroke: a single-center retrospective study

**DOI:** 10.1007/s00423-026-03978-8

**Published:** 2026-02-03

**Authors:** Bin Zheng, Min Li, Ningning Zhang, Rongguo Wang, Tao Wu, Chunxiao Wang

**Affiliations:** 1 Department of Neurosurgery, The Second Affiliated Hospital of Shandong First Medical University, Shandong, 271000 China; 2 Department of Medical Insurance, The Second Affiliated Hospital of Shandong First Medical University, Shandong, 271000 China; 3 Department of Medical, The Second Affiliated Hospital of Shandong First Medical University, Shandong, 271000 China; 4 Department of Pharmacy, Shandong First Medical University (Shandong Academy of Medical Sciences), Taian Campus, Taian, Shandong, 271016 China

**Keywords:** Cranioplasty, Decompressive craniectomy, Postoperative complications, Risk factors, Titanium mesh

## Abstract

**Background and objectives:**

Persistent controversy surrounds the identification of risk factors contributing to postoperative complications and unfavorable neurological prognoses following cranioplasty (CP) after decompressive craniectomy (DC). This study sought to assess these postoperative outcomes in individuals undergoing 3D titanium mesh CP due to traumatic brain injury (TBI) or hemorrhagic stroke, as well as to determine predictors linked to postoperative complications and suboptimal neurological recovery.

**Methods:**

A retrospective, single-center analysis was performed on patients undergoing 3D titanium mesh CP after DC. The primary outcome measure encompassed postoperative complications occurring within 12 months post-CP. The secondary endpoint involved the Glasgow Outcome Scale score assessed at 12 months following CP. We examined various parameters to identify predictors associated with postoperative complications and unfavorable neurological outcomes.

**Results:**

A total of 118 male patients (66%) were included, with trauma accounting for DC in 123 cases (68%). The overall incidence of postoperative complications was 45%, while poor neurological outcomes were observed in 30% of cases. Intraoperative dural depression during CP was correlated with an increased likelihood of postoperative epidural effusion; a time interval exceeding three months between DC and CP was associated with a heightened risk of hydrocephalus; bilateral CP demonstrated an elevated risk of wound dehiscence and infection. For poor GOS outcomes, pre-DC GCS score and DC due to stroke were identified as contributing factors, though no association was found with surgical timing.

**Conclusion:**

Early 3D titanium mesh CP, performed within three months after DC in TBI or stroke patients, appears to be a safe procedure without an increased incidence of postoperative complications or poor neurological prognosis. Conversely, delayed CP exceeding three months post-DC may increase the hydrocephalus’s likelihood. Patients experiencing intraoperative dural depression during CP should be closely monitored for the potential development of postoperative epidural effusion. Additionally, those undergoing bilateral CP are at greater risk for wound dehiscence and infection. Compared to individuals with TBI-induced DC, stroke patients undergoing CP tend to exhibit poorer neurological recovery.

## Introduction

Decompressive craniectomy (DC) is a frequently employed neurosurgical procedure aimed at alleviating intracranial hypertension that remains unresponsive to medical management by excising a portion of the skull. This surgical method predominantly involves the removal of a large unilateral bone flap, although bilateral partial bone flap excision may be warranted in certain clinical scenarios. It has been extensively utilized in managing patients experiencing severe elevations in intracranial pressure due to traumatic brain injury (TBI), stroke, or other critical neurological conditions, particularly in those who have already progressed to brain herniation. By creating essential space and time for subsequent therapeutic interventions, DC has been associated with enhanced survival outcomes [1]. Following the resolution of cerebral edema post-DC, the resultant skull defect lacks the necessary cranial protection, rendering it vulnerable to atmospheric pressure variations and positional shifts. These alterations influence cerebrospinal fluid (CSF) hydrodynamics and cerebral perfusion, potentially exacerbating neurological impairment. Prolonged absence of cranial reconstruction or the presence of extensive skull defects may contribute to the development of sinking flap syndrome [2], a condition characterized by deficits in motor function, cognition, and language, with severe manifestations posing life-threatening risks. Furthermore, psychological disturbances and difficulties in social interaction may also arise. Consequently, cranioplasty (CP) is routinely undertaken after DC, not only to restore the normal cranial contour for aesthetic purposes but also to shield brain tissue from secondary damage and facilitate neurological functional recovery.

Up to the present, debate persists concerning the determinants influencing postoperative complications and adverse neurological outcomes in patients undergoing skull repair, particularly those treated with 3D titanium mesh CP. In this study, clinical data were retrospectively gathered from individuals who underwent CP following DC due to craniocerebral trauma or hemorrhagic stroke, with a follow-up period of 12 months to monitor postoperative complications and neurological outcomes. This investigation was conducted to identify potential predictors of postoperative complications and unfavorable clinical outcomes, as well as to assess whether the timing of surgery impacts postoperative complications and neurological prognosis.

## Methods

### Data collection

**Fig. 1:**
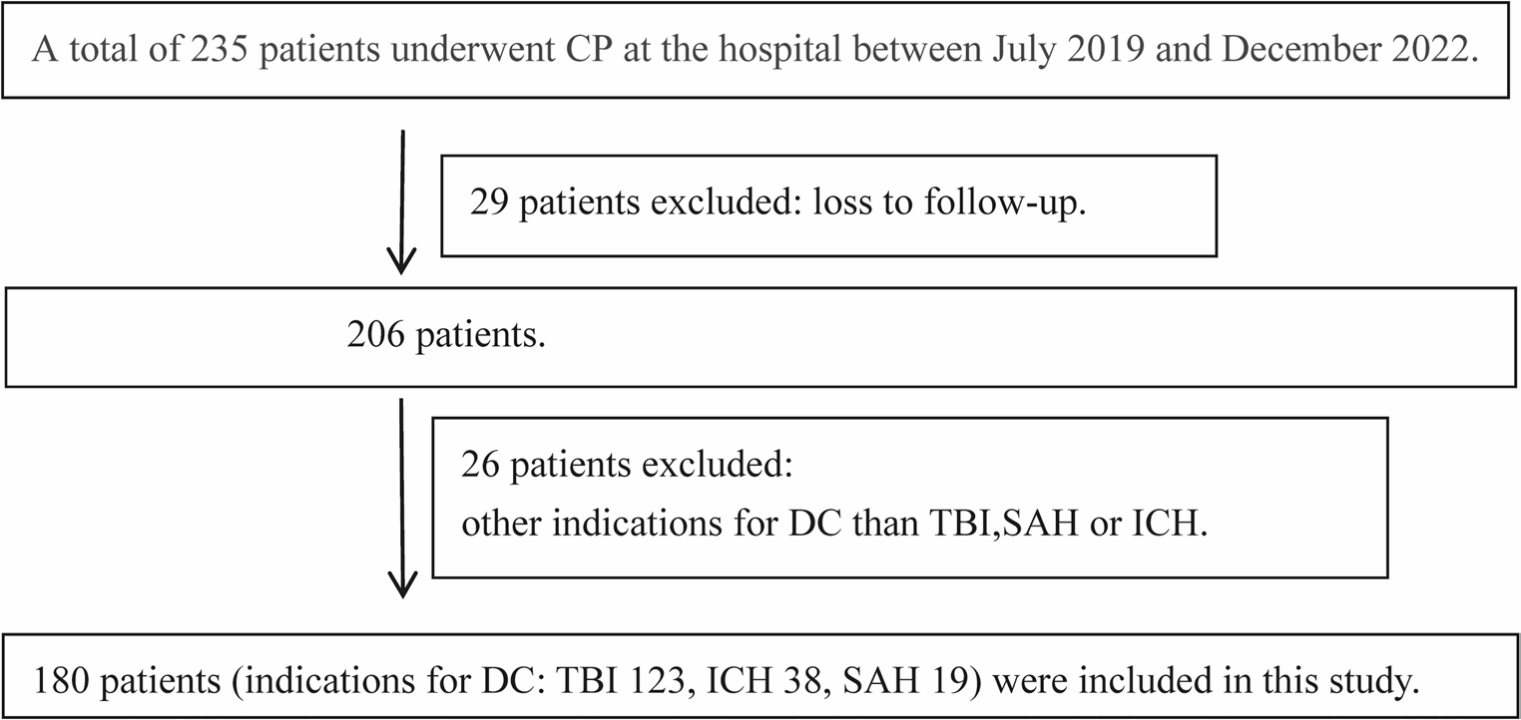
The flow chart illustrates the selection process of CP patients. CP, cranioplasty; DC, decompressive craniectomy; ICH, intracerebral hemorrhage; SAH, subarachnoid hemorrhage; TBI, traumatic brain injury

This single-center retrospective cohort study was performed per the ethical principles outlined by the World Medical Association (Declaration of Helsinki) and received approval from the institutional ethics committee. Due to the retrospective study design, the requirement for additional informed consent was waived. Clinical data were obtained from 180 patients who underwent CP at the Second Affiliated Hospital of Shandong First Medical University between July 2019 and December 2022 (Fig. 1). The inclusion criteria encompassed the following: (1) individuals who had undergone DC due to stroke or TBI, with the stroke category incorporating cases of subarachnoid hemorrhage (SAH) and intracerebral hemorrhage (ICH); (2) patients who had received 3D titanium mesh CP. The exclusion criteria were defined as follows: (1) cases in which DC was performed for reasons other than TBI or stroke, such as tumors or ischemic stroke; (2) individuals under the age of 13; (3) absence of computed tomography (CT) imaging within one week before CP or within 24 h post-CP; (4) contraindications for surgical intervention; and (5) loss to follow-up. Baseline characteristics, clinically relevant parameters, CT imaging findings, surgical details, postoperative complications, and neurological outcomes were documented. Follow-up assessments were carried out via outpatient visits or telephone interviews, concluding either upon patient mortality or at least 12 months post-CP, with follow-up durations ranging from 3 to 36 months. The primary endpoint was the occurrence of postoperative complications within 12 months following CP, while the secondary endpoint was the Glasgow Outcome Scale (GOS) score at 12 months postoperatively.

### Surgical methods

**Fig. 2:**
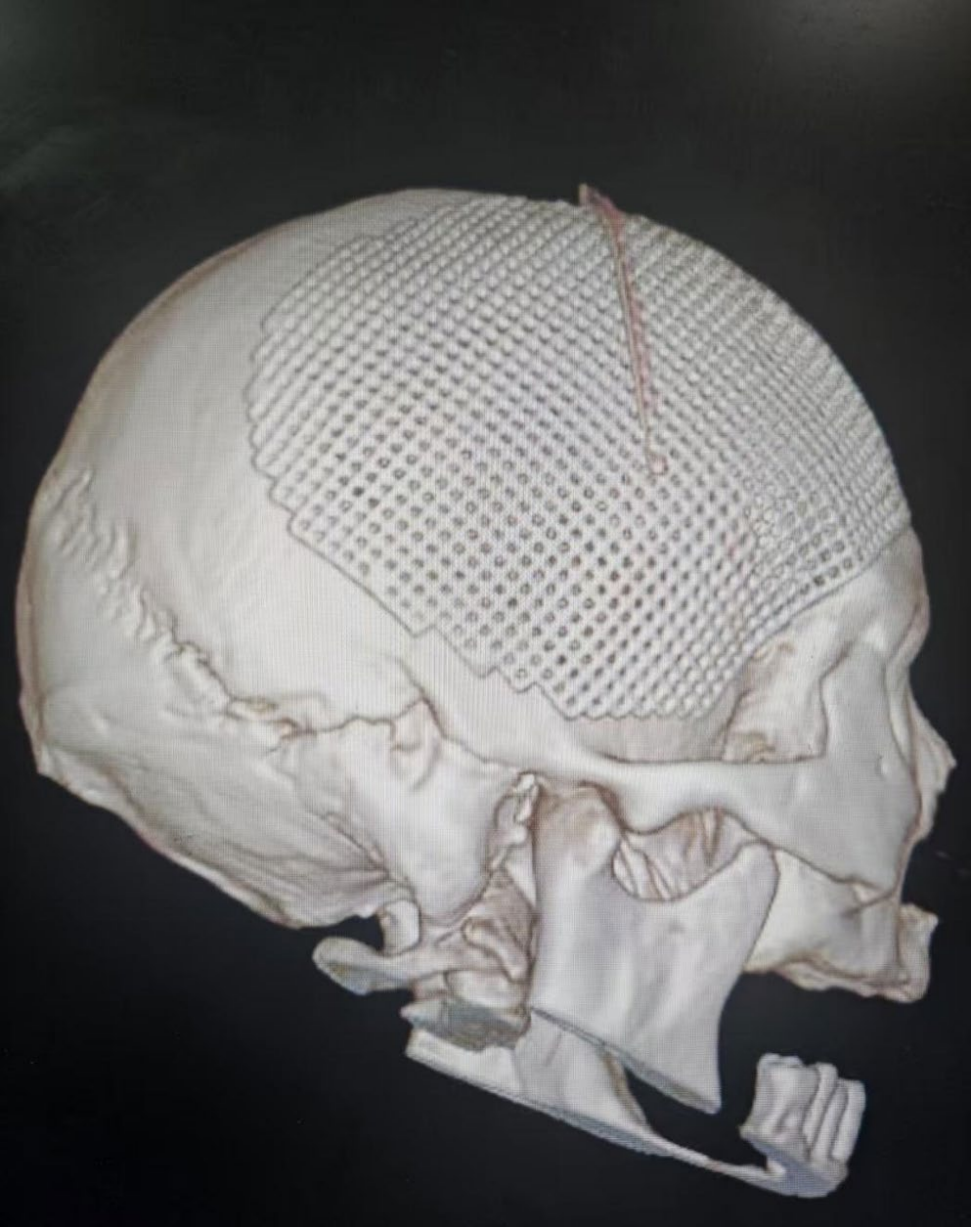
Cranial CT was re-examined within 24 h postoperatively (Reconstructed image)

Standardized clinical treatment protocols were implemented for patient management, with CP being performed by experienced neurosurgeons. Before skull reconstruction, 3D titanium mesh was individually tailored based on the patient’s three-dimensional CT imaging results. All procedures were conducted through the original incision under general anesthesia. The skin-muscle flap was carefully dissected, with the dura mater serving as the boundary, and every effort was made to preserve dural integrity. In cases where dural tears occurred, watertight suture repair was performed. The skull defect area was fully exposed to the margins of the bone window, and titanium pins were utilized to secure the titanium mesh in place. The dura mater was suspended and affixed to the titanium mesh, while the temporal muscle was sutured and anchored externally. Following meticulous hemostasis, a drainage tube was positioned beneath the scalp, external to the titanium mesh, and exited through the incision. The scalp was then sutured in multiple layers. To minimize the risk of postoperative infection, prophylactic antibiotics were administered. Cranial CT was re-examined within 24 h postoperatively (Fig. 2), and the drainage tube was removed within 48 h. Sutures were taken out 8 to 10 days following surgery.

### Observation indicators

1. Assessment variables: Several parameters were evaluated, including patient gender, age, pre-DC CT imaging findings such as basal cistern compression and midline shift, pupillary reflexes prior to DC, unilateral or bilateral skull defects, defect size, the interval between DC and CP, operative duration, intraoperative blood loss, dural positioning during CP, and a history of hypertension or diabetes.

Absence of pupillary reflex was characterized by non-reactivity in one or both pupils.

The skull defect area was determined using the formula 3.14 × long semi-axis × short semi-axis and was classified into two categories: small (< 140 cm²) or large (> 140 cm²). In cases of bilateral DC, the total defect area was calculated as the sum of both sides.

The pre-DC basal cistern condition was categorized as either normal or compressed.

The extent of midline shift before DC was measured at the maximal displacement point of the septum pellucidum on CT scans and was classified as either exceeding or not exceeding 5 mm.

The dural level during CP was assessed based on its positional relationship with the surrounding bone margin following the complete dissection of the myocutaneous flap from the dura during CP. A “dural depression” was defined as a condition where the dural surface was positioned below the surrounding bone margin during CP, whereas a “non-dural depression” referred to cases where the dural level remained at or above the bone margin.

The interval between DC and CP was stratified into two groups: the early group, which included patients undergoing CP within three months post-DC, and the late group, which comprised those whose CP was performed beyond three months after DC.

(2) The postoperative complications assessed encompassed new-onset epilepsy, epidural fluid collection, postoperative hemorrhage, wound dehiscence, infection, hydrocephalus, implant failure, and overall complications.

Surgical site infection was characterized by symptomatic or clinically apparent incisional infection.

**Fig. 3:**
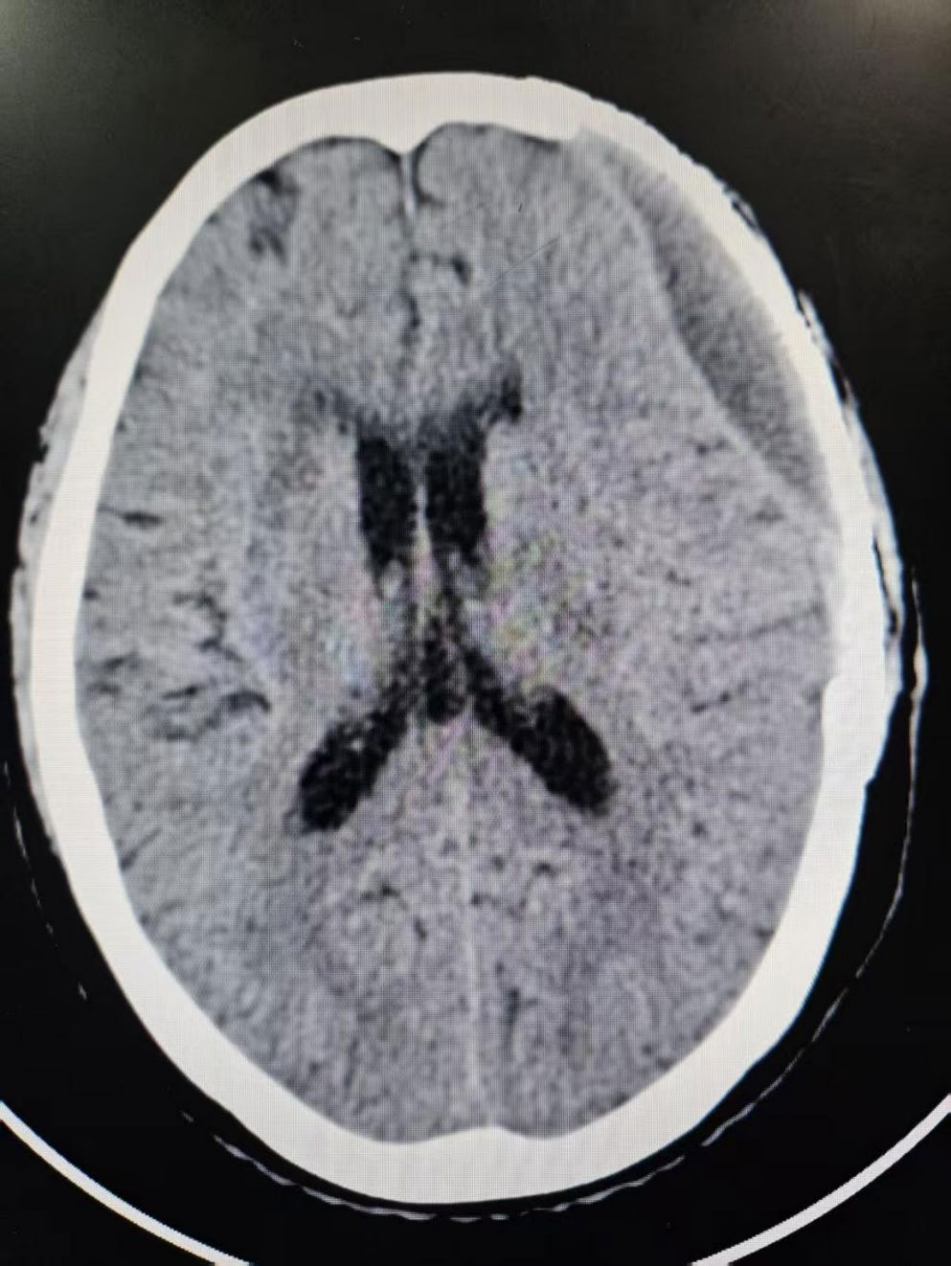
Epidural fluid collection (EFC) was identified based on low-density fluid in the epidural space on brain CT scans following CP

Epidural fluid collection (EFC) was identified based on low-density fluid in the epidural space on brain CT scans following CP (Fig. 3).

Postoperative hemorrhage included epidural hematoma, subdural hematoma, and intracerebral hemorrhage.

Hydrocephalus was diagnosed when ventricular enlargement occurred post-CP, necessitating permanent CSF diversion. In all cases of hydrocephalus, ventriculoperitoneal (V-P) shunt placement with adjustable pressure valves was performed. The accurate diagnosis of hydrocephalus was sometimes hindered by neurological deficits and impaired consciousness in certain patients, complicating the integration of clinical symptoms with radiological findings.

Implant failure was defined as the removal of the titanium mesh due to rejection reactions, wound dehiscence, implant exposure, or the need for reoperation owing to severe complications.

Patients experiencing one or more complications following CP were categorized within the overall complication group for statistical analysis of overall complications.

(3) Evaluation of neurological outcomes. The Glasgow Coma Scale (GCS) was employed to assess neurological status prior to DC upon admission [3], while the GOS was utilized for evaluation at the final follow-up [4]. The classification of outcomes was as follows: 1 point: death; 2 points: Vegetative state, characterized by an unconscious condition in which only fundamental physiological responses are preserved; 3 points: Severe disability, where patients remain conscious but suffer from profound neurological impairment, rendering them incapable of independent living; 4 points: Moderate disability, wherein some neurological deficits persist but do not interfere with essential self-care activities; 5 points: Good recovery, no significant neurological dysfunction, able to return to work or study. Poor neurological outcome was defined as a GOS score of 1–3.

### Statistical analysis

All statistical analyses were conducted utilizing SPSS 28.0. Continuous variables were denoted as mean ± SD, whereas categorical variables were denoted as percentages. The χ^2^ test was employed for categorical variables, while the Mann-Whitney U test was applied to assess continuous variables. To identify potential risk factors associated with overall complications, various complication subgroups, and unfavorable neurological outcomes, both univariate and multivariate logistic regression analyses were performed. Within the univariate logistic regression analysis, a*P*-value threshold of 0.1 was regarded as statistically significant, whereas for all other statistical evaluations, significance was determined at *P*-values < 0.05.

## Results

Table 1 presents the baseline characteristics of patients categorized based on the presence of postoperative complications. A total of 180 patients were included in this study, comprising 118 males (65.56%) and 62 females (34.44%). The cohort’s mean age was 51.37 ± 10.77 years, with a median age of 53 years (interquartile range [IQR]: 45–58). The predominant reason for DC was trauma, accounting for 68.33%, while bilateral surgery constituted 10%. Among the patients, 47% underwent early CP, whereas 53% received late CP. The median time interval (IQR) between DC and CP was 98 days (71–132 days). Specifically, in the early CP group, the median interval was 54 days (range: 33–90 days), whereas in the late CP group, the median interval was 4.5 months (range: 3–36 months). The mean duration of CP surgery was 144 ± 45.91 min. The mean volume of intraoperative blood loss during CP was 141 ± 84.81 mL. In the study cohort, 81 patients were classified into the complication group, while 99 patients were in the non-complication group, with no statistically significant differences observed in baseline characteristics between the two groups (*P* > 0.05).

**Table 1:** Comparison of demographic and surgical details classified by the presence or absence of postoperative complications

	Complication (−) (*n* = 99)	Complication (+) (*n* = 81)	*P*-value
Age(yrs)	49.92 ± 11.68	53 ± 9.29	0.097
Gender			0.156
Male	60	58	
Female	39	23	
Cause of DC			1.00
Trauma	68	55	
Stroke	31	26	
Defect laterality			0.078
Unilateral	93	69	
Bilateral	6	12	
Skull defect area(cm²)			0.653
>140 cm²	79	68	
< 140 cm²	20	13	
Operation time(mins)	142.74 ± 44.11	147.04 ± 48.18	0.595
Intraoperative blood loss(ml)	138.33 ± 80.75	145.31 ± 89.89	0.696
Hypertension			0.876
Yes	36	28	
No	63	53	
Diabetes			0.506
Yes	11	12	
No	88	69	
Time interval between DC and CP(months)			0.653
<3 months	48	36	
>3 months	51	45	
Dural depression			0.064
Yes	5	11	
No	94	70	

A total of 81 patients (36.3%) developed complications, with incidence rates detailed in Table 2. Collinearity analysis was conducted for all variables, including gender, age, causes of DC, CP timing, and unilateral or bilateral cranial defects, revealing no collinearity among these factors. The outcomes of the collinearity analysis for all assessed variables are displayed in Table 3.

**Table 2:** Number and percentage of complications

Complication type	*n*(%)
Overall complications	81 (45.0%)
Epidural fluid collection	31 (17.2%)
Hydrocephalus	16 (8.9%)
Postoperative new-onset seizures	20 (11.1%)
Wound dehiscence	9 (5.0%)
Surgical site infection	6 (3.3%)
Implant failure	5 (2.8%)
Postoperative hemorrhage	12 (6.7%)

**Table 3:** Collinearity analysis

Variable	Tolerance	Variance inflation factor
Gender	0.942	1.061
Age	0.879	1.138
TBI as cause of DC	0.491	2.037
DC-CP interval > 3 months	0.768	1.301
Dural depression	0.892	1.122
Hypertension	0.476	2.099
Diabetes	0.879	1.137
Bilateral CP	0.831	1.204
Operation duration	0.655	1.527
Intraoperative blood loss	0.656	1.525
Skull defect area >140 cm²	0.902	1.109

All non-collinear variables were incorporated into the logistic regression model. Binary univariate logistic regression analysis examined postoperative complications (Table 4). Based on the univariate analysis, no significant associations were identified for postoperative new-onset epilepsy or postoperative hemorrhage, whereas other postoperative complication subgroups exhibited statistically significant risk factors. After accounting for potential confounders, multivariate analysis was performed on postoperative complication subgroups, including variables with *P* < 0.1 (Table 5). The findings indicated that dural depression during CP demonstrated a significant association with the risk of EFC (*P* < 0.001). Additionally, a time interval exceeding 3 months between DC and CP was markedly linked to hydrocephalus (*P* = 0.041). Bilateral skull repair was found to be markedly associated with wound dehiscence (*P* = 0.049) and infection (*P* = 0.035). However, no significant associations were observed between overall complications and age, dural depression during CP, or bilateral skull repair.

**Table 4:** Results of univariate analysis for postoperative complications

Factors	Wound dehiscence	Epidural fluid collection	Hydrocephalus	Postoperative new-onset seizures	Implant failure	Postoperative hemorrhage	Infection	Overall complications
Gender	0.184	0.269	0.778	0.350	0.065	0.933	0.116	0.220
Age	0.991	0.168	0.678	0.833	0.443	0.366	0.164	0.048
TBI as cause of DC	0.536	0.729	0.106	0.241	0.686	0.898	0.929	0.910
Dural depression	0.999	<0.001	0.998	0.853	0.999	0.944	0.999	0.054
Diabetes	0.393	0.982	0.458	0.311	0.998	0.636	0.998	0.460
Hypertension	0.151	0.674	0.212	0.133	0.473	0.648	0.908	0.802
Bilateral CP	0.029	0.219	0.233	1.000	0.462	0.842	0.078	0.059
Operation time	0.720	0.333	0.267	0.306	0.869	0.531	0.515	0.531
Intraoperative blood loss	0.925	0.012	0.725	0.343	0.819	0.168	0.811	0.583
Cranial defect area>140 cm²	0.758	0.728	0.998	0.318	0.226	0.539	0.347	0.475
DC-CP interval > 3 months	0.891	0.545	0.029	0.751	0.763	0.406	0.511	0.589

**Table 5:** Results of multifactorial logistic regression analysis for specific complications

Complications	Factors	β	se	Wals	*P*-value	OR	95% CI
Wound dehiscence	Bilateral CP	2.114	1.073	3.882	0.049	8.285	1.011–67.893
Epidural fluid collection	Dural depression	2.339	0.644	13.193	<0.001	10.376	2.936–36.667
Hydrocephalus	DC-CP interval> 3 months	1.583	0.775	4.173	0.041	4.869	1.066–22.229
Implant failure	Gender	2.211	1.262	3.071	0.080	9.127	0.770–108.227.770.227
Infection	Bilateral CP	1.716	0.812	4.458	0.035	5.560	1.131–27.329
Overall complications	Age	0.028	0.016	3.049	0.081	1.028	0.997–1.061
	Dural depression	0.778	0.601	1.680	0.195	2.178	0.671–7.068
	Bilateral CP	0.923	0.594	2.415	0.120	2.517	0.786–8.063

A univariate analysis was conducted on predictive variables associated with poor prognosis in patients following CP, encompassing eight clinically relevant factors, including gender, age, and GOS score prior to DC. Variables with *P* < 0.1 were subsequently incorporated into the multivariate logistic regression analysis. The findings demonstrated that predictive factors for poor prognosis after CP included GCS prior to DC (*P* = 0.007, odds ratio [OR] = 0.843) and trauma as the underlying cause of DC (*P* = 0.027, OR = 2.277) (Table 6).

**Table 6:** Univariate and multivariate logistic regression analysis of predictive factors and timing for poor outcomes after CP

Factors	*P*-value of univariate analysis	*P*-value of multivariate analysis and adjusted OR value		95%C.I.
Gender	0.374			
Age	0.595			
GCS score before DC	<0.001	0.007	OR: 0.843	0.745–0.953
Absence of pupillary reflex before DC	0.004	0.401	OR:1.400	0.639–3.066
TBI as cause of DC	0.041	0.027	OR:0.439	0.211–0.913
Basal cistern compression	0.021	0.381	OR:0.671	0.275–1.638
Midline shift > 5 mm	0.017	0.173	OR: 0.573	0.258–1.277
DC-CP interval > 3 months	0.172			

## Discussion

CP has become a standard surgical intervention for patients who have undergone DC and is widely accepted by both medical institutions and patients. However, the optimal material for skull repair remains uncertain [5, 6]. Commonly utilized materials for reconstruction include autologous bone, polyetheretherketone (PEEK), and titanium mesh. Among these, autologous bone transplantation is frequently associated with complications such as bone flap absorption and infection. Despite their favorable properties, PEEK implants are hindered by high production costs, with the price in China estimated to be approximately 5–9 times higher than that of titanium mesh, limiting their widespread use. Although titanium mesh presents certain drawbacks, such as thermal conductivity and imaging artifacts, it also possesses notable advantages, including high strength, biological inertness, and relatively lower manufacturing costs. In our department, as well as in most regions of China, titanium mesh continues to be the preferred choice for skull defect repair following craniectomy. Furthermore, advancements in production technology, along with the integration of computer-assisted 3D modeling, have enhanced the precision of skull contour restoration, leading to improved cosmetic outcomes. Therefore, this study examined the risk factors associated with postoperative complications and unfavorable neurological outcomes in patients who underwent 3D titanium mesh CP at our institution, offering relevant recommendations.

### Postoperative complications

Recent research findings suggest that the incidence of complications following CP varies between 5% and 55% [7, 8]. This considerable fluctuation in reported complication rates can be attributed to discrepancies in the definition of complications, variations in the types of complications considered, differences in surgical protocols, diversity in cranial repair materials, and the administration of antibiotics. The complication rate observed in this study aligns with this reported range.

This study aimed to determine potential risk factors associated with complications following CP. Ultimately, three distinct risk factors were identified for different postoperative complication subgroups. Dural concavity observed during CP was linked to an elevated risk of postoperative EFC. A time interval exceeding 3 months between DC and CP was found to be associated with an increased likelihood of hydrocephalus. Additionally, bilateral skull reconstruction was correlated with a higher risk of wound dehiscence and infection.

In this study, EFC was identified as the most frequently occurring postoperative complication.Previous studies have rarely focused on or emphasized EFC, possibly due to its spontaneous absorption in most cases, leading to favorable prognoses and infrequent occurrences of severe neurological impairment. However, some patients present with symptoms such as headache, infection, and wound dehiscence. In cases where EFC accumulates in large volumes, interventions such as subcutaneous drainage tube placement and reoperation are required [9–11], resulting in extended hospital stays and increased financial burdens. In the present study, patients diagnosed with EFC were effectively managed through fluid aspiration followed by pressure bandaging with elastic bandages or corticosteroid administration for 3–7 days, with no instances requiring secondary surgical intervention. The exact pathogenesis of EFC remains uncertain. Studies on autologous bone CP have identified epidural air bubbles and dural calcification as potential predictive factors for EFC formation following CP [12, 13]. In this cohort, CT examinations conducted within 24 h after CP revealed that some patients exhibited both epidural air bubbles and EFC. However, these bubbles were non-tensional and resolved rapidly in subsequent CT follow-ups. A retrospective analysis by Zhang et al. involving 340 CP patients reported an EFC formation rate of 34.41%, with skull defect size and intraoperative dural suspension recognized as predictive factors for EFC development [13, 14]. Other studies have proposed that EFC occurrence is linked to CP materials, with PEEK implants demonstrating a higher likelihood of inducing EFC compared to titanium mesh [6, 15]. Shields et al. suggested that postoperative EFC associated with PEEK materials might represent an allergic reaction, with successful treatment observed following corticosteroid administration [16]. Additionally, Jeong et al. hypothesized that CSF leakage resulting from intraoperative dural injury contributes to EFC formation [11]. Currently, limited clinical research has explored the effect of intraoperative dural depression on EFC. Some institutions have utilized preoperative skin depression in the skull defect area as a predictive indicator of postoperative EFC [17–19]. In this study, the dural level was evaluated in relation to the surrounding bone edge during CP to minimize the influence of subcutaneous fluid accumulation and preoperative positional changes. Intraoperative dural depression was determined to be an independent risk factor for postoperative EFC. This may be because under general anesthesia, with the surgical side typically positioned superiorly, persistent dural depression signifies poor brain compliance. Following surgery, inadequate brain tissue re-expansion may result in a persistent cavity between the subcutaneous tissue and dura, which remains unapproximated even with intraoperative dural suspension, ultimately leading to EFC formation. This suggests that physical factors contribute to the development of EFC. Furthermore, EFC formation is also influenced by individual patient characteristics and allergic responses to various repair materials.

This study further identified bilateral CP as an independent risk factor for postoperative wound dehiscence and infection. Wound dehiscence occurring after CP is frequently accompanied by infection, which serves as a primary contributor to implant failure [20]. The involvement of the frontal sinus during bilateral CP may introduce a potential source of infection, thereby elevating the risk of postoperative complications. Additionally, bilateral CP is associated with prolonged surgical exposure and extended incision length, both of which contribute to impaired wound healing and increased susceptibility to infection, findings that align with those reported by Fallatah et al. [21]. Previous studies have documented postoperative infection rates following CP ranging from 0.8% to 16% [8, 22, 23]. In the present study, the postoperative infection rate was observed to be 3.3%. However, comparisons across studies are constrained by inconsistencies in the definition of infection, variations in CP materials, and differences in the administration of prophylactic antibiotics prior to surgery [24]. For instance, Foster et al. determined that patients who underwent CP with calcium phosphate cement exhibited markedly lower rates of wound infection and CSF leakage compared to those who received titanium mesh CP [25]. Implant failure commonly results from factors such as implant exposure, infection, impaired wound healing, and rejection reactions. Multiple variables contribute to implant failure, including advanced age, compromised nutritional status, a history of diabetes, excessive incision tension, and the selection of different repair materials. To mitigate or address implant failure, Dambrino et al. proposed that stricter postoperative infection control, the involvement of plastic surgeons in the procedure, and individualized treatment plans for patients experiencing complications may lead to improved clinical outcomes [26–28].

In this study, it was observed that a time interval exceeding 3 months between DC and CP was associated with an increased risk of hydrocephalus. Yang et al. demonstrated that patients undergoing early CP exhibited a lower incidence of ventricular enlargement compared to those who underwent late CP [29], a finding consistent with the present results. Other investigations have similarly reported that among patients whose DC was exclusively caused by TBI, early CP correlated with a reduced occurrence of hydrocephalus [21, 30–32]. In the current study cohort, TBI patients comprised 68.33% of the sample, and no statistically significant difference was identified in hydrocephalus incidence between the TBI and stroke groups following CP (*P* = 0.106). The development of hydrocephalus may be influenced by factors such as SAH, age, lower GOS scores, and extensive skull defects [33]. Although the precise mechanism remains uncertain [34–36], it is evident that alterations in CSF circulation dynamics disrupt the previous balance between CSF production and absorption. It is hypothesized that hydrocephalus leads to establishing a “new equilibrium,” determined by either an adjusted intracranial pressure or total CSF volume, rendering patients intolerant to these changes and subsequently leading to clinical symptoms. In this cohort, the majority of hydrocephalus shunting procedures were conducted more than 3 months after DC. One possible explanation is that post-DC hydrocephalus represents a form of chronic communicating hydrocephalus, which does not necessitate urgent intervention. Clinically, when ventricular enlargement is observed in conjunction with skull defects, the standard treatment strategy prioritizes addressing the skull defect before determining the necessity of a V-P shunt based on the patient’s hydrocephalus status post-CP. This treatment sequence aligns with the consensus established by Corrado et al. [37]. Among previously treated patients, some exhibited either a reduction in ventricular size or an improvement in hydrocephalus symptoms following CP, thereby negating the need for a V-P shunt. This phenomenon may partially explain why most V-P procedures were performed more than 3 months post-DC. However, conflicting perspectives exist. Morton et al.’s single-center retrospective study reported that performing skull repair beyond 90 days after DC markedly lowered the incidence of hydrocephalus [38]. Given the ongoing debate surrounding this issue, further prospective studies and multicenter comparisons are necessary to clarify these findings.

Currently, no authoritative guidelines or consensus has been established regarding whether the timing of CP following DC affects postoperative complications. Generally, CP is performed within 3 to 6 months after DC. Certain studies propose that the occurrence of postoperative complications is influenced by CP timing, advocating for early CP once brain edema has subsided, with early CP commonly defined as occurring within 3 months post-DC [20, 39]. Investigations exploring even earlier CP have also been conducted, with Chun et al. reporting that CP performed within one month after DC is both safe and effective, without an increased risk of surgical complications [40]. Conversely, some studies have found no statistically significant differences in postoperative complication rates between early and late CP [41, 42], while research by Goedemans et al. suggests that early CP may be associated with a higher incidence of postoperative complications [43]. In the present study, late CP was found to elevate the risk of hydrocephalus, whereas no significant differences were detected between the early and late CP groups in other complication subgroups. Additionally, operation duration (*P* = 0.561) and intraoperative blood loss (*P* = 0.841) did not markedly differ between the two groups, suggesting comparable surgical safety. Furthermore, no definitive risk factors for overall patient complications were identified.

### Neurological outcomes

The assessment of patients’ neurological outcomes was conducted using the GOS, which was selected for its simplicity, widespread applicability in research, and practicality in follow-up evaluations. However, a limitation of the GOS lies in its pseudo-ordinal nature, and in numerous studies, outcome classification has been dichotomized, thereby further diminishing the score’s sensitivity. Through univariate and multivariate logistic regression analyses, pre-DC GCS score (OR: 0.843, 95% confidence interval [95%CI]: 0.745–0.953) and TBI as the underlying cause of DC (OR: 2.277, 95%CI: 1.096–4.731) were identified as risk factors for poor GOS prognosis. Moreover, no significant association was found between surgical timing and poor GOS outcomes. The effect of surgical timing on neurological recovery remains a subject of debate. Aloraidi et al. reported that neurological outcomes in early and late CP patients were nearly identical, with assessments based on the GOS and the modified Rankin scale indicating no significant influence of CP timing [41], findings that align with those of the present study. However, contradictory perspectives exist. A systematic review and meta-analysis involving 528 patients demonstrated that early CP markedly improved neurological outcomes [44]. Similarly, Safi et al. suggested that CP facilitates neurological recovery, with early CP potentially enhancing this process [31]. Beyond surgical timing, postoperative neurological prognosis is influenced by multiple factors [45], closely linked to the cause of DC, the patient’s pre-DC condition, surgical details, postoperative rehabilitation, and other variables [46, 47], necessitating a multidisciplinary and multi-departmental approach to treatment. Among these factors, the etiology of DC plays a particularly significant role. It was observed that stroke as the cause of DC was a risk factor for poor GOS prognosis. The possible reasons are as follows: Firstly, the bleeding sites in stroke patients often occur in critical areas such as the brain stem, thalamus, and basal ganglia. Patients requiring surgery generally have a larger amount of bleeding, which can damage important neural conduction tracts and nuclei, leading to severe residual functional impairment. Secondly, compared to TBI patients, stroke patients tend to be older, with comorbidities such as hypertension and diabetes, resulting in poorer physical function and immunity, which increases the risk of complications and subsequently affects prognosis. The mechanisms underlying neurological improvement after CP may be associated with increased cerebral blood flow, stabilization of intracranial pressure, alterations in brain wave activity, and enhanced CSF circulation [48–51]. However, definitive evidence remains lacking as to whether neurological recovery results from CP surgery itself or the natural self-repair mechanisms of the nervous system over time following craniocerebral injury.

### Strengths and limitations

In contrast to most clinical studies on patients undergoing titanium mesh CP, this single-center retrospective study analyzed a relatively large cohort of 180 cases. A detailed characterization of complication types was provided, and an extensive array of risk factors was considered. However, several limitations should be acknowledged. First, the retrospective single-center design may have introduced selection bias. Second, the study population was relatively homogeneous, as only patients undergoing CP due to TBI or hemorrhagic stroke were included without further stratification of these subgroups. Third, the influence of rehabilitation on postoperative complications and neurological outcomes was not examined, given the variability in rehabilitation center types, treatment approaches, and durations. Fourth, limitations related to incomplete follow-up data and insufficient follow-up duration persist, particularly considering that neurological recovery is a prolonged process necessitating extended monitoring at consistent intervals. Future research should aim to overcome these limitations through larger-scale prospective studies incorporating more diverse patient cohorts.

## Conclusion

Three risk factors associated with postoperative complications following CP were identified. Dural concavity observed during CP surgery was linked to an elevated risk of postoperative EFC. A time interval exceeding three months between DC and CP was associated with an increased likelihood of hydrocephalus. Additionally, bilateral skull repair heightens the risk of wound dehiscence and infection. For poor GOS outcomes, pre-DC GCS score and DC due to stroke were identified as contributing factors, though no association was found with surgical timing. Early titanium mesh repair after DC for stroke or TBI is considered a safe procedure, with no increase in postoperative complications or adverse neurological outcomes, whereas delayed repair may elevate the risk of hydrocephalus. Patients exhibiting intraoperative dural concavity should undergo postoperative monitoring for EFC, while those undergoing bilateral skull repair face a heightened risk of wound dehiscence and infection. Furthermore, patients with stroke as the underlying etiology demonstrate poorer neurological outcomes following CP in comparison to those with TBI. A patient-specific approach is recommended for individuals with varying skull defects, emphasizing a comprehensive evaluation of preoperative and intraoperative factors rather than relying on a singular predictive measure.

## Data Availability

All the data used in the current work can be made available on request.

## Abbreviations

CP: Cranioplasty
CSF: Cerebrospinal fluid
CT: Computed tomography
DC: Decompressive craniectomy
EFC: Epidural fluid collection
GCS: Glasgow Coma Scale
GOS: Glasgow Outcome Scale
ICH: Intracerebral hemorrhage
TBI: Traumatic brain injury
